# The relationship of circulating fibroblast growth factor 21 levels with pericardial fat: The Multi-Ethnic Study of Atherosclerosis

**DOI:** 10.1038/s41598-019-52933-9

**Published:** 2019-11-11

**Authors:** Arsenios Magdas, Jingzhong Ding, Robyn L. McClelland, Matthew A. Allison, Philip J. Barter, Kerry-Anne Rye, Kwok Leung Ong

**Affiliations:** 1 Lipid Research Group, School of Medical Sciences, University of New South Wales, Sydney, NSW Australia; 2 School of Medicine, University of Notre Dame, Sydney, NSW Australia; 3 Sticht Center on Aging, Wake Forest University School of Medicine, Winston-Salem, NC United States; 4 Department of Biostatistics, University of Washington, Seattle, WA United States; 5 Department of Family Medicine and Public Health, University of California San Diego, La Jolla, CA United States

**Keywords:** Predictive markers, Cardiovascular biology, Obesity, Risk factors

## Abstract

Previous small studies have reported an association between circulating fibroblast growth factor 21 (FGF21) levels and pericardial fat volume in post-menopausal women and high cardiovascular disease (CVD) risk patients. In this study, we investigated the relationship of FGF21 levels with pericardial fat volume in participants free of clinical CVD at baseline. We analysed data from 5765 men and women from the Multi-Ethnic Study of Atherosclerosis (MESA) with both pericardial fat volume and plasma FGF21 levels measured at baseline. 4746 participants had pericardial fat volume measured in at least one follow-up exam. After adjusting for confounding factors, ln-transformed FGF21 levels were positively associated with pericardial fat volume at baseline (β = 0.055, p < 0.001). When assessing change in pericardial fat volume over a mean duration of 3.0 years using a linear mixed-effects model, higher baseline FGF21 levels were associated with higher pericardial fat volume at baseline (2.381 cm^3^ larger in pericardial fat volume per one SD increase in ln-transformed FGF21 levels), but less pericardial fat accumulation over time (0.191 cm^3^/year lower per one SD increase in ln-transformed FGF21 levels). Cross-sectionally, higher plasma FGF21 levels were significantly associated with higher pericardial fat volume, independent of traditional CVD risk factors and inflammatory markers. However, higher FGF21 levels tended to be associated with less pericardial fat accumulation over time. Nevertheless, such change in pericardial fat volume is very modest and could be due to measurement error. Further studies are needed to elucidate the longitudinal relationship of baseline FGF21 levels with pericardial fat accumulation.

## Introduction

Pericardial fat is located between the external surface of the parietal pericardium and the internal surface of the mediastinum^1^. It includes epicardial fat and paracardial fat. A recent study has shown a significant association between excessive pericardial fat volume, especially fat in the atrioventricular groove, and coronary artery disease^2^. Additionally, a previous Multi-Ethnic Study of Atherosclerosis (MESA) has reported the association of pericardial fat with risk of coronary heart disease (CHD), independent of classical cardiovascular risk factors such as body mass index (BMI), blood lipids, fasting glucose, and C-reactive protein (CRP) levels^3^. A more recent study of the MESA cohort reported a significant association between pericardial fat and poorer CVD prognosis including higher annualised risk of all cause death, heart failure, all-cause CVD, coronary heart disease and stroke^4^.

FGF21 regulates glucose and lipid metabolism in multiple organs such as the liver, heart, skeletal muscle, pancreas and others^5–7^. Several recent animal studies have shed light on the beneficial effects of FGF21 administration on glucose and lipid metabolism, including decreased body weight and improved lipid profiles^5–7^. However, in humans, circulating FGF21 levels are often elevated in different pathophysiological conditions such as metabolic syndrome, obesity, dyslipidemia, insulin resistance, type 2 diabetes, non-alcoholic fatty liver disease and coronary artery disease^5^. There is strong evidence suggesting a significant association between elevated serum FGF21 levels and the development of atherosclerosis, myocardial ischemia, coronary heart disease, cardiac hypertrophy, and diabetic cardiomyopathy^6,8^.

Small clinical studies have suggested a positive association of circulating FGF21 levels with epicardial or pericardial fat volume in post-menopausal women and people at high CVD risk^9,10^. However, it is not known whether such an association is relevant to men and healthy people, and whether there are any sex or racial/ethnic differences in the association of plasma FGF21 levels with pericardial fat volume. Therefore, in the present study, the relationship of FGF21 levels and pericardial fat was investigated using a large-scale well-established cohort of participants, free of clinically apparent CVD.

## Materials and Methods

### Participants

The MESA is a cohort of 6814 adult men and women recruited from six United States communities (Baltimore, MD; Chicago, IL; Forsyth County, NC; Los Angeles County, CA; New York, NY, and St. Paul, MN) between July 2000 and August 2002. At baseline exam 1, all participants were aged between 45–84 years, from one of four major racial/ethnic groups (Caucasian, African American, Hispanic American and Chinese American) and had no clinical evidence of CVD. The participants underwent four additional follow up assessments over a period of 10 years (exams 2, 3, 4, and 5 in 2002–2004, 2004–2005, 2005–2008 and 2010–2012, respectively). Informed written consent was obtained from all participants and the study was approved by the institutional review boards at all participating centres (Johns Hopkins Medicine Institutional Review Board, Wake Forest University Health Sciences Institutional Review Board, Northwestern University Institutional Review Board, University of California Los Angeles Institutional Review Board, Columbia University Medical Center Institutional Review Board, University of Minnesota Institutional Review Board, University of Washington Institutional Review Board, and University of Vermont Institutional Review Board). The analysis of FGF21 levels was also approved by the University of New South Wales Sydney Human Research Advisory Panel. The study was conducted in adherence with all principles of the Declaration of Helsinki. The objectives, design and protocol of the study have been described previously^11^.

Of the 6814 participants at baseline, data on pericardial fat volume at the baseline exam were available on 6785 participants, 5765 of whom also plasma FGF21 levels measured.

### Pericardial fat measurement

Pericardial fat measurements were obtained from thoracic computed tomography (CT) scans. Three field centres used an electro-beam CT scanner while the other 3 field centres utilised a multidetector row helical CT scanner. The resulting CT scans were analysed for pericardial fat volume as described previously^3^. In brief, the CT slices within 15 mm above and 30 mm below the superior extent of the left main coronary artery were analysed by three different experienced CT analysts. This region of the heart was selected as it includes the pericardial fat located around all the main proximal coronary arteries (left main coronary, left anterior descending, right coronary, and circumflex arteries). The volume analysis software (GE HealthCare, Waukesha, WI) was used to discern fat from other tissues according to a threshold of −190 to −30 Hounsfield units. Pericardial fat volume was defined as the sum of all voxels containing fat. This measure of pericardial fat volume was previously found to be highly correlated with total volume of pericardial fat volume^12^. CT scans from a random sample of 80 MESA participants were reread and the intraclass correlation coefficients of intra-reader and inter-reader reliability were 0.99 and 0.89, respectively, for pericardial fat^3^.

### FGF21 measurement

Venous blood samples were collected after a 12-hour fast using standardized venipuncture procedures. Plasma FGF21 levels were measured by enzyme-linked immunosorbent assay kits (Antibody and Immunoassay Services, University of Hong Kong, Hong Kong) at baseline^13,14^. Briefly, plasma (60 μl) was diluted 1:1 (v/v) with assay diluent and analyzed together with quality controls according to manufacturer’s instructions. The intra-assay and inter-assay coefficients of variation were < 10%. Patient identity was masked for all samples analysed.

### Other variables of interest

Standardized questionnaires were used to ascertain information regarding participant age, gender, smoking, education, alcohol use, race/ethnicity, physical activity, medical history and medication. Education level was defined as high school, less than high school and more than high school. Cigarette smoking was defined as never, former, and current smoking. Physical activity was summarized as the total number of reported hours of moderate and vigorous activities per week, multiplied by metabolic equivalent level as previously described^15^.

Height and weight measurements were taken with participants wearing light clothing and no shoes. BMI was calculated as the weight (in kilograms) divided by height (in meters squared). Hip and waist circumferences were obtained using standard flexible tape measures. A Dinamap model Pro 100 automated oscillometric sphygmomanometer (Critikon) was used to take three measurements of sitting resting blood pressure. The average of the last two blood pressure readings was used for analysis. Hypertension was defined as use of antihypertensive medications, blood pressure ≥140/90 mm Hg or previous diagnosis of hypertension. Diabetes was defined as use of glucose-lowering medications or fasting glucose ≥126 mg/dL. Estimated glomerular filtration rate (eGFR) was calculated using the creatinine-based Chronic Kidney Disease Epidemiology Collaboration equation^16^. The homeostasis model assessment index of insulin resistance (HOMA-IR) was calculated according to the updated computer model as described previously^17^. The lipid profile, and CRP and interleukin-6 (IL-6) levels were measured as previously described^18,19^.

### Statistical analysis

Data analysis was performed using SPSS 24 (IBM, Armonk, NY) or STATA 14.0 (StataCorp, College Station, TX). Data were presented as mean (SD) or percentage (number). For variables with a skewed distribution, data were presented as median (interquartile range). Distributions of demographic data, CVD risk factors, and pericardial fat volume at baseline were compared across FGF21 quartiles among all the participants. Age, sex, BMI, race/ethnicity and other variables that showed an increasing or decreasing trend with FGF21 levels after adjusting for age, sex, and race/ethnicity (p < 0.1) were used as covariates in subsequent multiple regression analyses. A multivariable linear regression model was used to investigate the cross-sectional association of plasma FGF21 levels with pericardial fat volume using robust standard error estimation after adjusting for age, sex, and ethnicity. In model 1, data were adjusted for demographic, socioeconomic and lifestyle factors, including age, sex, race/ethnicity, education, smoking, pack-years of smoking, current alcohol use and physical activity. In model 2, data were further adjusted for established CVD risk, including BMI, diabetes, hypertension, HOMA-IR, high-density lipoprotein (HDL) cholesterol, triglycerides, eGFR, and use of lipid-lowering medication, which have been found to correlate with FGF21 levels in previous studies^5,9,10,13,14^. As FGF21 has anti-inflammatory effects^8^ and pericardial fat can secrete pro-inflammatory cytokines^1^, data were further adjusted for circulating inflammatory biomarkers, including CRP and IL-6 in model 3, in order to assess whether these inflammatory biomarkers can mediate the association.

No multi-collinearity issue was detected as assessed by the variance inflation factors (VIF). All VIF were < 3. The non-linear relationship of FGF21 levels with pericardial fat volume at baseline was assessed using regression splines. In this analysis, no non-linear relationship was found between FGF21 levels and pericardial fat volume at baseline.

Although participants attended up to four additional follow-up visits over a 10-year period, pericardial fat volume was not measured in exam 5. Pericardial fat volume was measured in 4515 out of the 5765 participants (78.3%) at either exam 2 or 3, and in 921 participants (16.0%) at exam 4. Therefore, a total of 4056 out of the 5765 participants (70.4%) had pericardial fat volume measured at only one follow-up exam, either at exam 2, 3, or 4. Only 690 (12.0%) participants had pericardial fat volume measured at two follow-up exams. We assessed the relationship of baseline FGF21 levels with longitudinal change in pericardial fat volume using a standard linear mixed-effects model^20^ among all participants, utilizing data on pericardial fat volume measured at all follow-up exams. In the linear mixed-effects model, we used random intercepts (representing baseline pericardial fat volume for each participant) and random slope (representing the change in pericardial fat volume per year) with adjustment for fixed effect of the same set of covariates as in the cross-sectional analysis. The linearity assumption was assessed by the inspection of diagnostic residual plots.

In all the analyses, we assessed whether there was any sex or racial/ethnic interaction, and p for interaction was estimated by including the interaction term in the regression models in the full sample after adjusting for the main effects of the covariates. In all regression analyses, replacement of BMI by waist-to-hip ratio and height in the adjustment model made little difference to the results (data not shown). A two-tailed p < 0.05 was considered statistically significant.

## Results

### Participant characteristics

The characteristics of the 5765 participants according to the quartiles of plasma FGF21 levels at baseline are shown in Table 1. Participants with higher plasma FGF21 levels were more likely to be older, female, Caucasian or Hispanic American, and current smoker, with lower education level. Additionally, participants with higher plasma FGF21 levels tended to have lower height, physical activity, HDL cholesterol and eGFR, but higher BMI, waist-to-hip ratio, triglycerides, HOMA-IR, IL-6 and CRP levels. They also had higher prevalence of diabetes and hypertension and were more likely to take lipid-lowering medications.

**Table 1:** Baseline characteristics of participants (n = 5765).

Characteristics	n	FGF21 level, pg/mL				P value
		Quartile 1	Quartile 2	Quartile 3	Quartile 4	
n	5765	1441	1443	1440	1441	
Age, years	5765	60.3 ± 10.2	62.6 ± 10.1	63.6 ± 10.1	64.2 ± 10.1	<0.001
Women, n (%)	5765	698 (48.4)	716 (49.6)	788 (54.7)	805 (55.9)	<0.001
Race/ethnicity, n (%)	5765					<0.001
Caucasian	2150	511 (35.5)	530 (36.7)	547 (38.0)	562 (39.0)	
African American	1661	478 (33.2)	431 (29.9)	382 (26.5)	370 (25.7)	
Hispanic American	1256	253 (17.6)	293 (20.3)	343 (23.8)	367 (25.5)	
Chinese American	698	199 (13.8)	189 (13.1)	168 (11.7)	142 (9.9)	
Education, n (%)	5744					<0.001
<High school	1048	208 (14.5)	248 (17.2)	292 (20.4)	300 (20.9)	
High school	2408	544 (37.9)	597 (41.4)	621 (43.4)	646 (44.9)	
>High school	2288	682 (47.6)	596 (41.4)	518 (36.2)	492 (34.2)	
BMI, kg/m^2^	5765	27.2 ± 5.0	27.8 ± 5.1	28.9 ± 5.7	29.4 ± 5.7	<0.001
Waist-to-hip ratio	5765	0.91 ± 0.08	0.93 ± 0.08	0.94 ± 0.08	0.95 ± 0.08	<0.001
Height, cm	5765	167.4 ± 9.6	166.9 ± 10.0	165.7 ± 10.4	165.2 ± 10.0	<0.001
Smoking, n (%)	5745					
Never	2889	764 (53.2)	725 (50.3)	713 (49.8)	687 (47.8)	0.009
Former	2128	514 (35.8)	548 (38.0)	533 (37.2)	533 (37.1)	
Current	728	157 (10.9)	168 (11.7)	185 (12.9)	218 (15.2)	
Pack-years of smoking	5683	8.8 ± 16.4	11.3 ± 20.8	11.6 ± 20.6	13.5 ± 23.7	<0.001
Current alcohol use, n (%)	5720	787 (55.2)	826 (57.6)	773 (54.2)	762 (53.1)	0.089
Physical activity (MET-hours/week)	5747	102.7 ± 94.2	98.6 ± 101.4	87.9 ± 92.2	89.0 ± 98.0	<0.001
Diabetes, n (%)	5755	118 (8.2)	173 (12.0)	198 (13.8)	249 (17.3)	<0.001
Hypertension, n (%)	5765	518 (35.9)	645 (44.7)	677 (47.0)	781 (54.2)	<0.001
Lipid-lowering medication, n (%)	5753	198 (13.8)	243 (16.9)	234 (16.3)	292 (20.3)	<0.001
LDL cholesterol, mg/dL	5686	117.0 ± 31.3	117.9 ± 31.8	117.4 ± 30.3	116.3 ± 32.7	0.563
HDL cholesterol, mg/dL	5755	53.4 ± 15.4	51.7 ± 14.9	50.2 ± 14.6	48.4 ± 14.1	<0.001
Triglycerides, mg/dL^*^	5758	89 (64–125)	105 (78–151)	121 (84–170)	136 (92–199)	<0.001
HOMA-IR^*^	5739	0.80 (0.59–1.14)	0.89 (0.64–1.30)	0.97 (0.71–1.42)	1.12 (0.75–1.62)	<0.001
IL-6, pg/mL^*^	5620	0.98 (0.67–1.56)	1.17 (0.75–1.84)	1.29 (0.85–2.00)	1.49 (0.96–2.29)	<0.001
CRP, mg/L^*^	5732	1.40 (0.62–3.20)	1.68 (0.83–4.00)	2.10 (0.91–4.45)	2.57 (1.12–5.36)	<0.001
eGFR, mL/min/1.73 m^2^	5755	80.5 ± 15.1	78.3 ± 15.5	77.2 ± 16.1	74.0 ± 17.9	<0.001
Pericardial fat at baseline volume, cm^3^	5765	65.5 ± 35.9	75.9 ± 39.2	86.0 ± 43.4	90.9 ± 43.6	<0.001

Data are expressed as mean ± SD, or n (%), or median (interquartile range). FGF21 quartiles 1, 2, 3, and 4 were defined as FGF21 levels of ≤81.2, 81.3–146.1, 146.2–245.4, and ≥245.5 pg/mL. Unadjusted *p* values were estimated by ANOVA for continuous variables and chi-square test for categorical variables respectively.

^*^P values were estimated using ln-transformed data.

### Association of FGF21 and pericardial fat volume at baseline

Cross-sectionally, FGF21 levels correlated positively with pericardial fat volume (Spearman correlation coefficient = 0.269, p < 0.001) and participants with higher plasma FGF21 levels were more likely to have larger pericardial fat volume (Table 1). In multivariable linear regression analysis, higher plasma FGF21 levels were significantly associated with larger pericardial fat volume at baseline after adjusting for demographic, socioeconomic and lifestyle factors, although the association was modest (Model 1, Table 2). Further adjustment for CVD risk factors attenuated the association, which still remained statistically significant (Model 2, Table 2). Further adjusting for inflammatory biomarkers still resulted in a significant association between plasma FGF21 levels and pericardial fat volume (Model 3, Table 2). In addition, there was a significant interaction by sex (p for interaction < 0.001), in which the association was more pronounced in men than in women (β* = *0.062 and 0.054 respectively). However, there was no significant interaction by race/ethnicity (p for interaction = 0.089).

**Table 2:** Association of ln-transformed FGF21 levels with pericardial fat volume at baseline.

Model	β	P value
1	0.180	<0.001
2	0.061	<0.001
3	0.055	<0.001
Men	0.062	<0.001
Women	0.054	<0.001

Regression coefficient (β) was estimated with SD as analytic unit, i.e. number of SD (41.8 cm^3^) difference in pericardial fat volume associated with a change of one SD (1.35) in ln-transformed FGF21 level).

Model 1: Adjusted for age, sex, race/ethnicity, education, smoking, pack-years of smoking, current alcohol use and physical activity.

Model 2: Further adjusted for BMI, diabetes, hypertension, ln-transformed HOMA-IR, HDL cholesterol, ln-transformed triglycerides, eGFR, and use of lipid-lowering medication.

Model 3: Further adjusted for ln-transformed CRP and ln-transformed IL-6.

FGF21 indicates fibroblast growth factor 21.

### Association of baseline FGF21 levels with change in pericardial fat volume

Among 5765 participants, 4746 participants had pericardial fat volume measured at least one follow-up exam with mean duration of 3.0 years with a range of 1.0–6.8 years. Table 3 shows the results on the association of baseline FGF21 levels with rate of change in pericardial fat volume using a linear mixed-effects model. Higher baseline FGF21 levels were associated with higher pericardial fat volume at baseline, which remained significant after adjusting for demographic, socioeconomic and lifestyle factors, CVD risk factors, and inflammatory biomarkers (2.381 cm^3^ larger in pericardial fat volume per one SD increase in ln-transformed FGF21 levels). Moreover, a higher baseline ln-transformed FGF21 levels were significantly associated with modestly less pericardial fat accumulation over time (0.191 cm^3^/year lower per one SD increase in ln-transformed FGF21 levels).

**Table 3:** Association of baseline FGF21 levels with rate of change in pericardial fat volume (n = 5765).

Model	Intercept	Slope
1	7.588 (0.503)^†^	−0.184 (0.080)^*^
2	2.631 (0.432)^†^	−0.192 (0.081)^*^
3	2.381 (0.437)^†^	−0.191 (0.082)^*^

The intercept estimate represents the regression coefficient (SE) for the association of FGF21 levels with pericardial fat volume at baseline. The slope estimate represents the difference (SE) in the rate of change in pericardial fat volume (cm^3^) per year per unit increment in FGF21. Data are expressed as per SD (1.35) increase in ln-transformed levels.

Model 1: Adjusted for age, sex, race/ethnicity, education, smoking, pack-years of smoking, current alcohol use and physical activity.

Model 2: Further adjusted for BMI, diabetes, hypertension, ln-transformed HOMA-IR, HDL cholesterol, ln-transformed triglycerides, eGFR, and use of lipid-lowering medication.

Model 3: Further adjusted for ln-transformed CRP and ln-transformed IL-6.

^*^P < 0.05 and ^†^*P* < 0.001.

## Discussion

In this present study, elevated circulating FGF21 levels were significantly associated with elevated pericardial fat volume cross-sectionally and in a multi-ethnic cohort of adults without a history of clinical cardiovascular disease. The association tended to be stronger in men than in women. When analyzing the change in pericardial fat volume over time using linear mixed-effects model, higher baseline FGF21 levels were found to be associated with less pericardial fat accumulation over time. However, it should be noted that the association of FGF21 levels with pericardial fat volume and its accumulation over time is modest only and may have limited clinical significance.

FGF21 shows beneficial effects in animal and clinical studies. FGF21 administration can improve glucose and lipid metabolism, including a decrease in body weight and an improvement in lipid profiles in obese and diabetic mice^7^. Furthermore, FGF21 also shows cardioprotective properties, such as lipid-lowering, anti-inflammatory and antioxidant effects in cell culture and animal studies^8^. In fact, FGF21 also exerts a cardioprotective effect post myocardial infarction through the activation of an adiponectin-dependent pathway^21^. In human clinical trials, administration of FGF21 analogs can reduce body weight and improve lipid profile in obese/overweight type 2 diabetic subjects^22,23^.

Despite the cardioprotective effect of FGF21, circulating FGF21 levels are often elevated in different metabolic disorders^5^. In human studies, circulating FGF21 levels correlate with BMI^24^ and are elevated in rodents with diet-induced obesity^25^. The elevation in FGF21 levels in these conditions, and in people with elevated pericardial fat volume in the present study, could be a compensatory protective response to the underlying metabolic stress, or due to FGF21 resistance^25^, in which impaired interactions of FGF21 with its receptor and downregulation of downstream signaling pathways requires supraphysiological doses of FGF21 to achieve its protective physiological function. In fact, it has been reported that epicardial adipose tissue can express FGF21 and the expression is increased in response to surgery-related inflammation and insulin resistance after cardiac surgery^26^.

The present study supports a significant cross-sectional association between FGF21 levels and pericardial fat volume. In a recent case-control study of 86 post-menopausal women, serum FGF21 levels were positively and significantly correlated with epicardial fat thickness in the obese women^9^. In that study, among different clinical and biochemical parameters including BMI, LDL cholesterol, HDL cholesterol, CRP and HOMA-IR, epicardial fat showed the strongest association with serum FGF21 levels^9^. In another recent cross-sectional study of 60 patients with coronary artery disease and 129 BMI-matched controls, higher serum FGF21 levels were independently associated with pericardial fat volume after adjusting for age, sex, BMI, triglycerides and HOMA-IR^10^. However, these studies have the limitation of small sample size and cross-sectional study design. In the present study, FGF21 levels were found to be associated with pericardial fat volume in a much larger, ethnically diverse population, even after adjusting for more CVD risk factors, including the pro-inflammatory markers, CRP and IL-6.

Previous studies using data from the MESA have reported the association of pericardial fat with coronary heart disease and poorer CVD outcomes^3,4^. In a recent study in mice, plasma FGF21 levels were reported to be an early predictive biomarker for the development of CVD risk factors such as insulin resistance, metabolic disturbance and cardiac FGF21 resistance^27^. Therefore, it is possible that elevated plasma FGF21 levels may predict pericardial fat accumulation, which is a well-established CVD risk factor. Moreover, in two small proof-of-concept trials, administration of synthetic FGF21 analogs decreased body weight and improved the lipid profile in obese\overweight patients with type 2 diabetes^22,23^. Therefore, it would be interesting to investigate whether FGF21 levels has a causal effect on pericardial fat accumulation. However, in the present study, we found that elevated plasma FGF21 levels were associated with less pericardial fat accumulation over time. Nevertheless, interpretation should be cautious as the change in pericardial fat volume was very modest and may have limited clinical significance. Moreover, we could not exclude the possibility that the findings on the changes in pericardial fat volume could be false positive due to measurement error or the phenomenon of regression to the mean^28^.

The present study suggests a possible sex difference in the association between FGF21 levels and pericardial fat volume at baseline. In previous studies, pericardial fat volume was found to be lower in women, than in men^29,30^. In a recent rat study, hepatic FGF21 expression levels were up-regulated by a chronic high-fat, high-fructose diet in male rats, but not in female rats due to suppression of FGF21 resistance by estrogen^31^. Further studies are needed to investigate whether estrogen may affect the expression and function of FGF21 in pericardial fat.

The present study has several strengths. Compared to previous studies on the relationship of FGF21 levels with pericardial fat or epicardial fat, the finding from the present study should be more robust due to its large sample size and ethnically diverse population. The present study also takes the advantage of a large well-characterised sample of participants apparently free of clinical CVD at the time of recruitment and the associated good quality data. There are also some limitations in this study. The major limitation is that the two components of pericardial fat: paracardial fat and epicardial fat, have not been separately assessed. Recent clinical evidence suggests that epicardial fat plays a more prominent role in CVD and its risk factors such as insulin resistance, metabolic syndrome and subclinical atherosclerosis, compared with pericardial fat^1^. However, to distinguish epicardial fat from paracardial fat, one must identify the pericardium which is often difficult to visualise, especially in lean individuals^32^. Moreover, pericardial fat volume was only measured in a sub-cohort in exams 2, 3 and 4, in which the mean duration between the baseline measurement and the last follow-up measurement was about 3 years and the changes in pericardial fat volume were small, which may lead to the modest association observed in this study. Another major limitation is that FGF21 levels were not measured at follow-up visits, thus it was not possible to assess how FGF21 levels changed relative to pericardial fat volume, and whether pericardial fat volume at baseline can predict change in FGF21 levels.

In conclusion, higher plasma FGF21 levels at baseline were associated with higher pericardial fat volume at baseline. Higher FGF21 levels tended to be associated with less pericardial fat accumulation over time, however the change in pericardial fat volume was very modest and could be due to measurement error. Further studies are needed to elucidate the longitudinal relationship of baseline FGF21 levels with pericardial fat accumulation and clarify the physiological role of FGF21 in pericardial fat accumulation.
